# Supplementary figures and images for: Genome-wide identification of new reference genes for RT-qPCR normalization in CGMMV-infected Lagenaria siceraria
Source: PeerJ. 2018 Oct 12;6:e5642. doi: 10.7717/peerj.5642 (PMC6188008; doi:10.7717/peerj.5642)

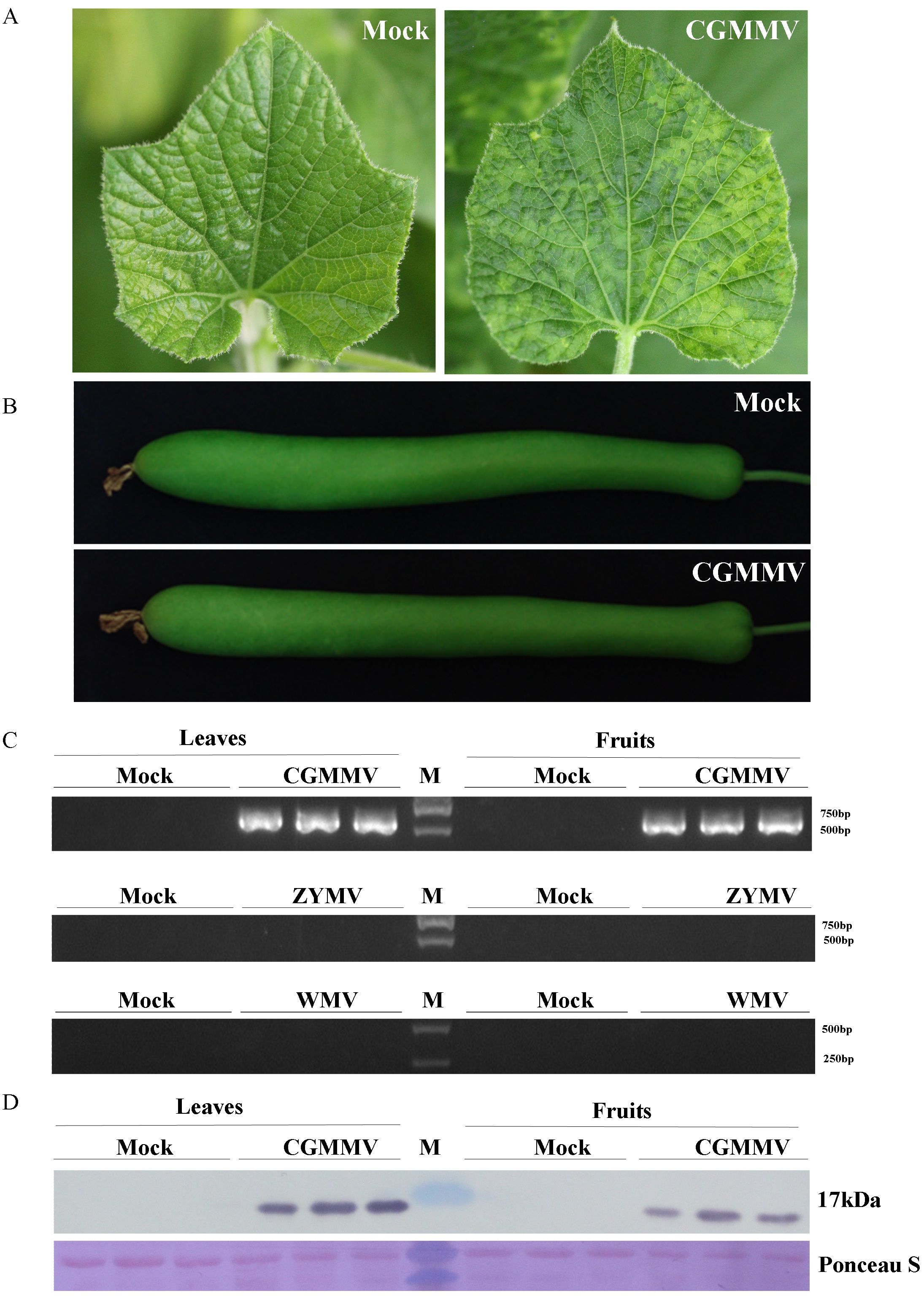

Supplement: Figure S1 — (A) The typical green mottle mosaic symptom on CGMMV-infected leaves 14 days after inoculation (right panel), while not symptom on mock leaves (left panel). (B) No obvious symptoms of virus on the fruit of CGMMV-infected bottle gourd (the bottom panel) and the mock fruits (the upper panel). (C) Detection of CGMMV (the upper panel), ZYMV (the middle panel) and WMV (the bottom panel) on L. siceraria by RT-PCR. (D) Detection of CGMMV by western blot. Photos by Chenhua Zhang. [file peerj-06-5642-s001.png]

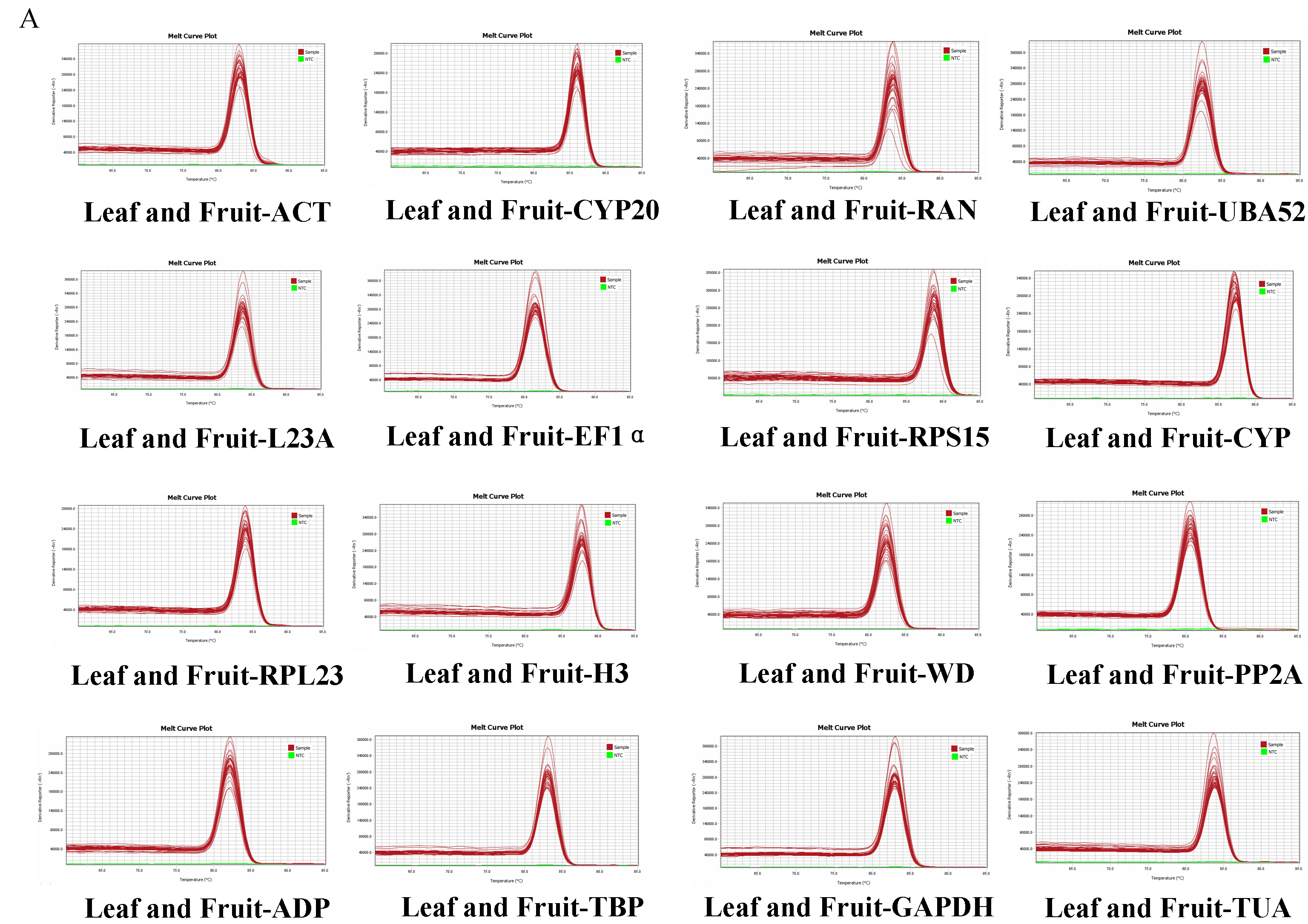

Supplement: Figure S2A — (A) Dissolution curves of candidate RGs on both leaf and fruit of bottle gourd. (B) Dissolution curves of candidate RGs on leaf of bottle gourd. (C) Dissolution curves of candidate RGs on fruit of bottle gourd. [file peerj-06-5642-s002.png]

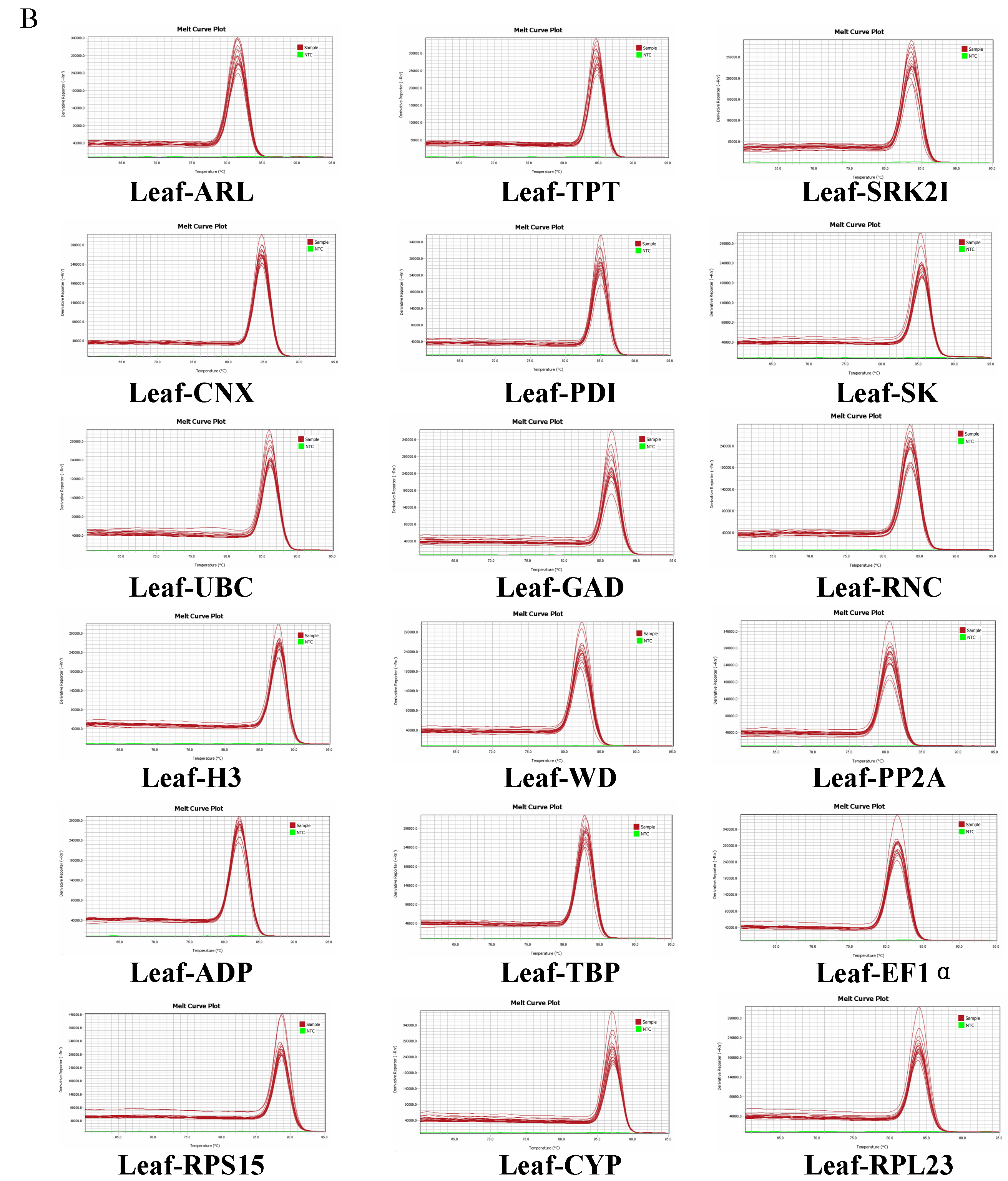

Supplement: Figure S2B — (A) Dissolution curves of candidate RGs on both leaf and fruit of bottle gourd. (B) Dissolution curves of candidate RGs on leaf of bottle gourd. (C) Dissolution curves of candidate RGs on fruit of bottle gourd. [file peerj-06-5642-s003.png]

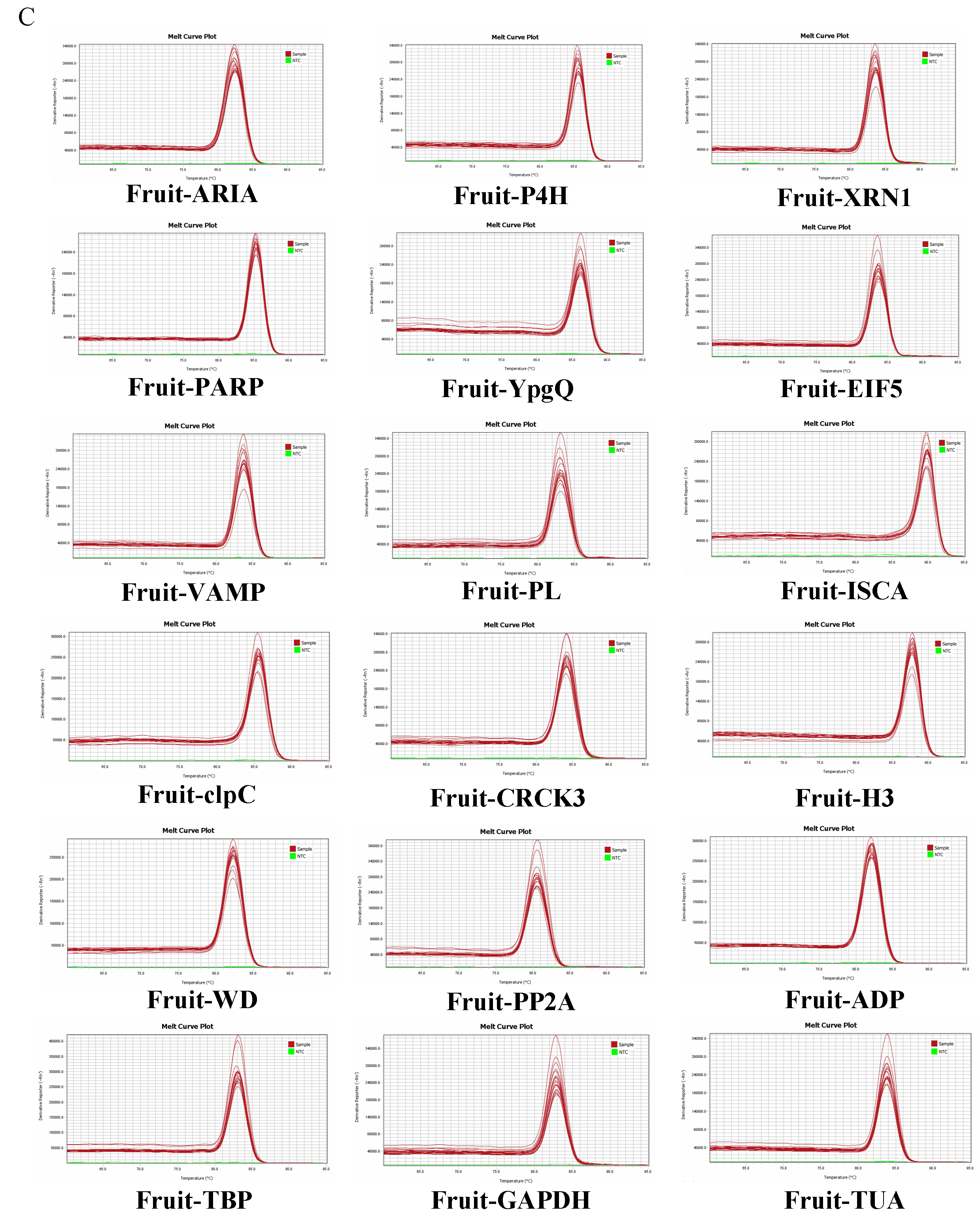

Supplement: Figure S2C — (A) Dissolution curves of candidate RGs on both leaf and fruit of bottle gourd. (B) Dissolution curves of candidate RGs on leaf of bottle gourd. (C) Dissolution curves of candidate RGs on fruit of bottle gourd. [file peerj-06-5642-s004.png]

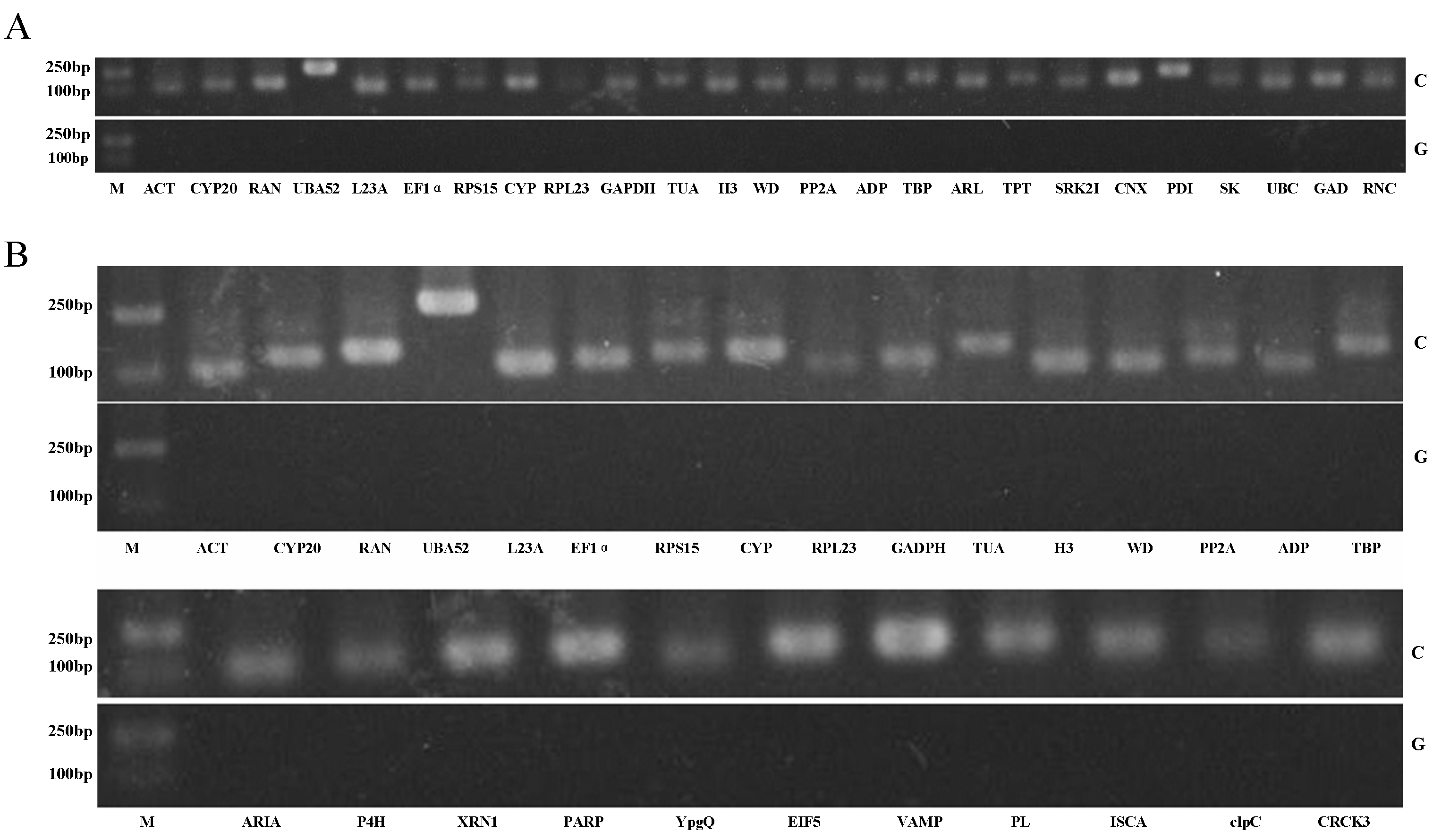

Supplement: Figure S3 — The upper line “C”, indicates the cDNA template, the bottom line “G”, indicates the gDNA template. “M” indicates the DNA ladder marker. (A) PCR amplification products of all RGs in L. siceraria leaf. (B) PCR amplification products of all RGs in L. siceraria fruit. [file peerj-06-5642-s005.png]

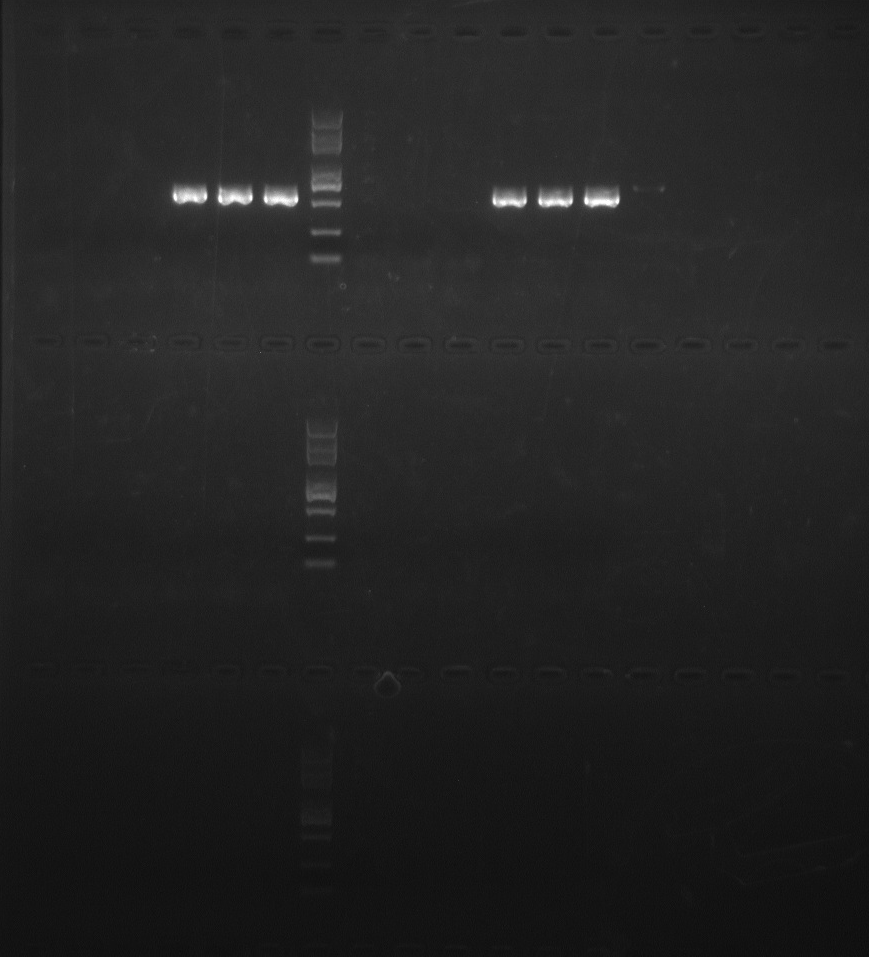

Supplement: Supplemental Information S2 [file peerj-06-5642-s016.zip › The raw photographs, electrophoretic gels and blots-1/Fig. S1 Detection of CGMMV ZYMV and WMV in systemic leaves and fruits of L. siceraria By RT-PCR-1.png]

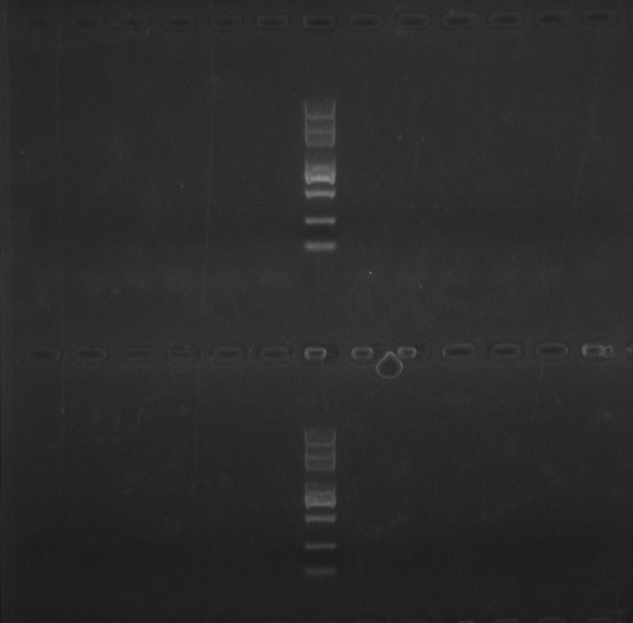

Supplement: Supplemental Information S2 [file peerj-06-5642-s016.zip › The raw photographs, electrophoretic gels and blots-1/Fig. S1 Detection of CGMMV ZYMV and WMV in systemic leaves and fruits of L. siceraria By RT-PCR-2.png]

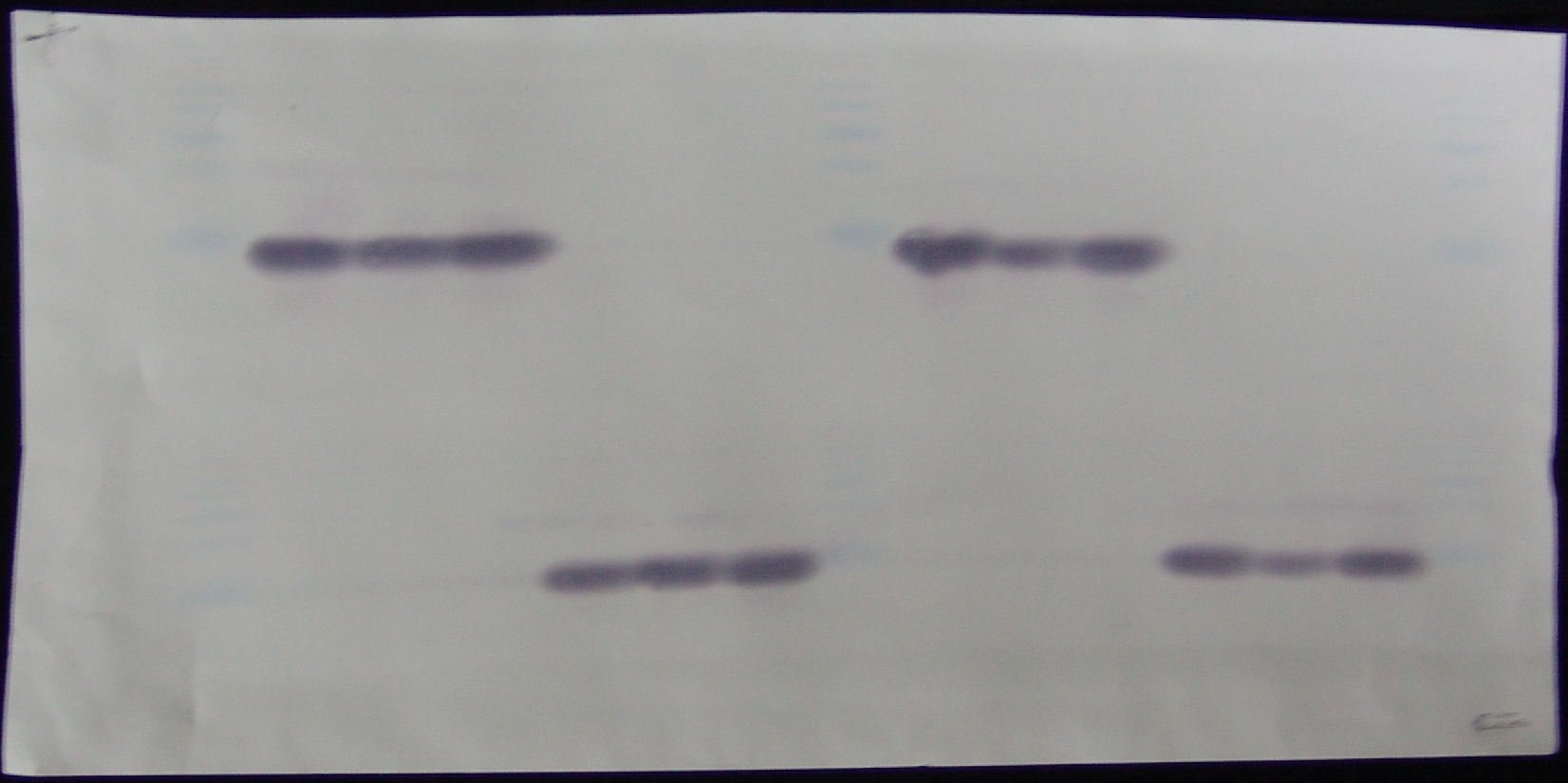

Supplement: Supplemental Information S2 [file peerj-06-5642-s016.zip › The raw photographs, electrophoretic gels and blots-1/Fig. S1 Detection of CGMMV in systemic leaves and fruits of L. siceraria By western blot 1.png]

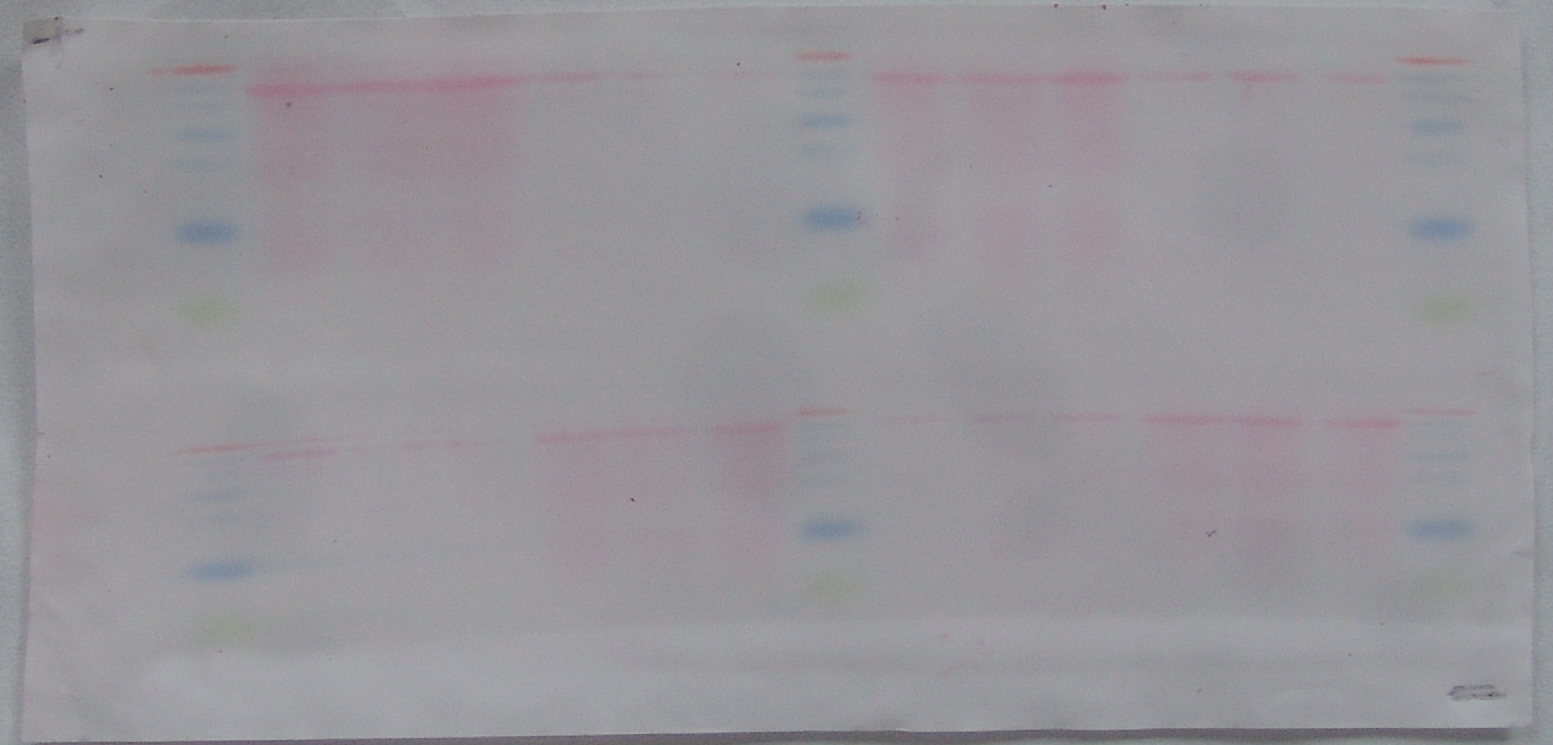

Supplement: Supplemental Information S2 [file peerj-06-5642-s016.zip › The raw photographs, electrophoretic gels and blots-1/Fig. S1 Detection of CGMMV in systemic leaves and fruits of L. siceraria By western blot 2.png]

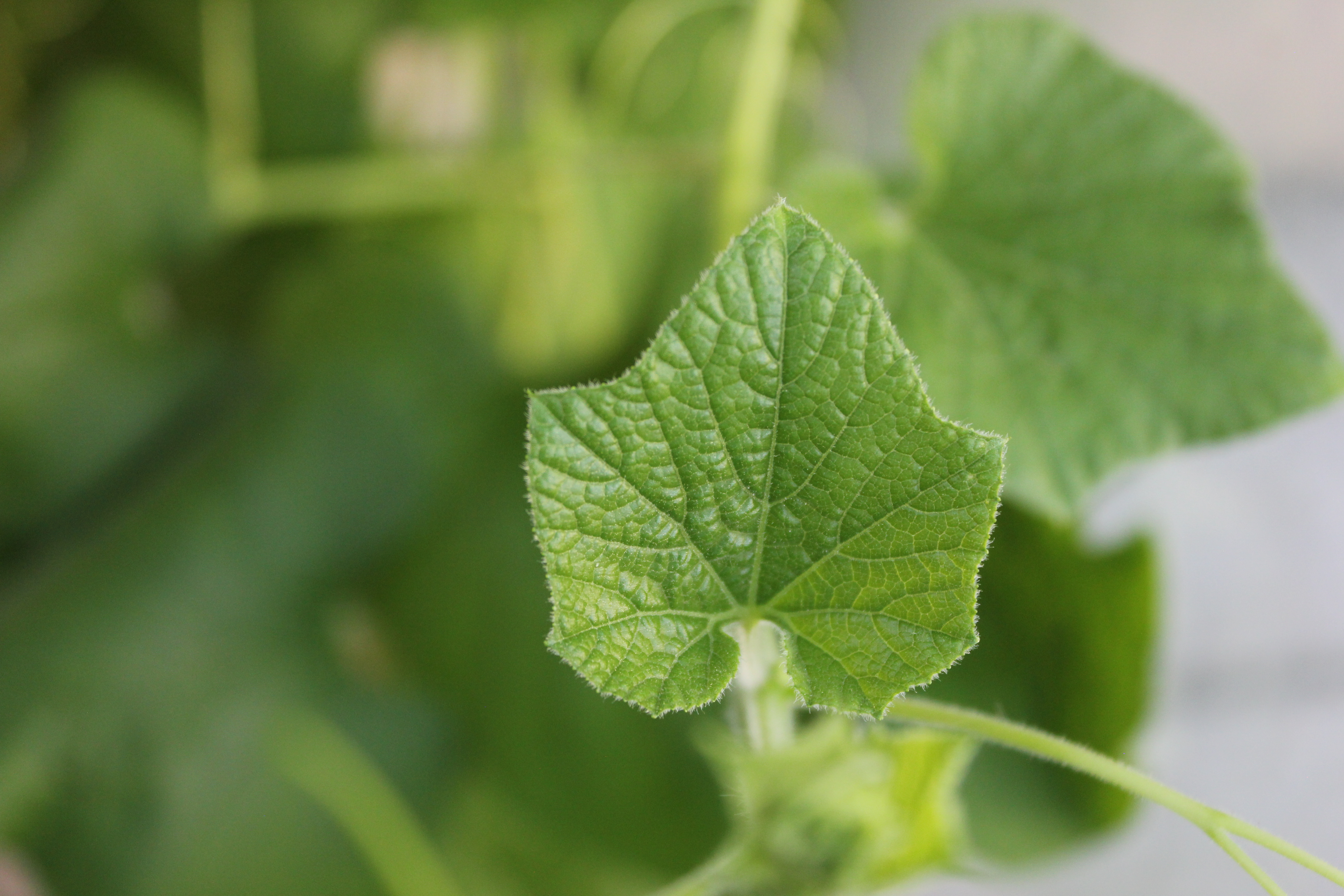

Supplement: Supplemental Information S2 [file peerj-06-5642-s016.zip › The raw photographs, electrophoretic gels and blots-1/Fig. S1 The healthy control bottle gourd leaf.png]

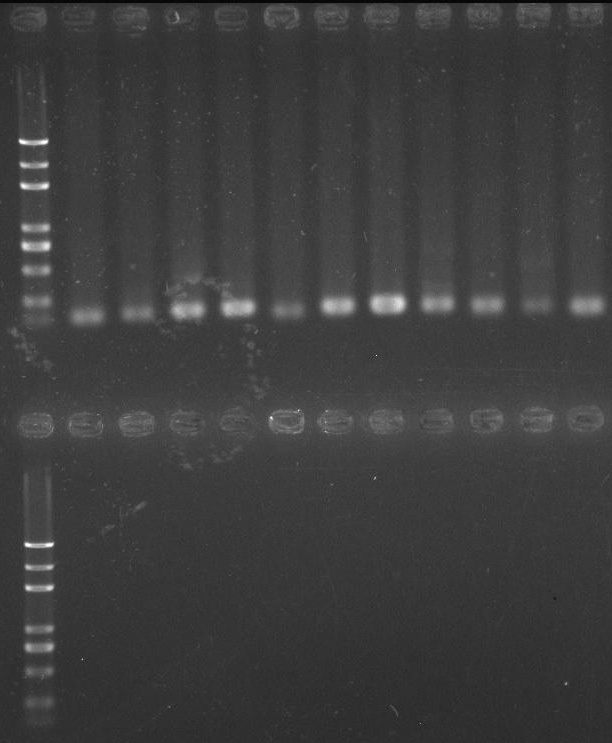

Supplement: Supplemental Information S2 [file peerj-06-5642-s016.zip › The raw photographs, electrophoretic gels and blots-1/Fig. S3 Polymerase chain reaction (PCR) amplification products of all reference genes in L. siceraria fruit in 2% agarose gel-2.png]

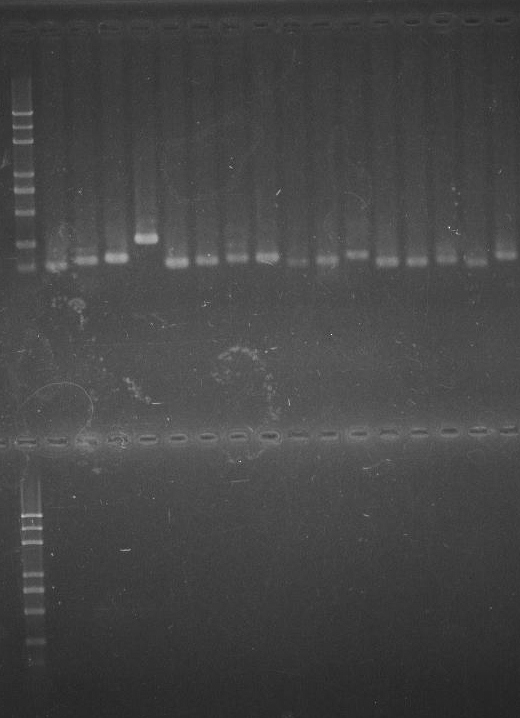

Supplement: Supplemental Information S2 [file peerj-06-5642-s016.zip › The raw photographs, electrophoretic gels and blots-1/Fig. S3 Polymerase chain reaction (PCR) amplification products of all reference genes in L. siceraria fruit in 2% agarose gel-3.png]

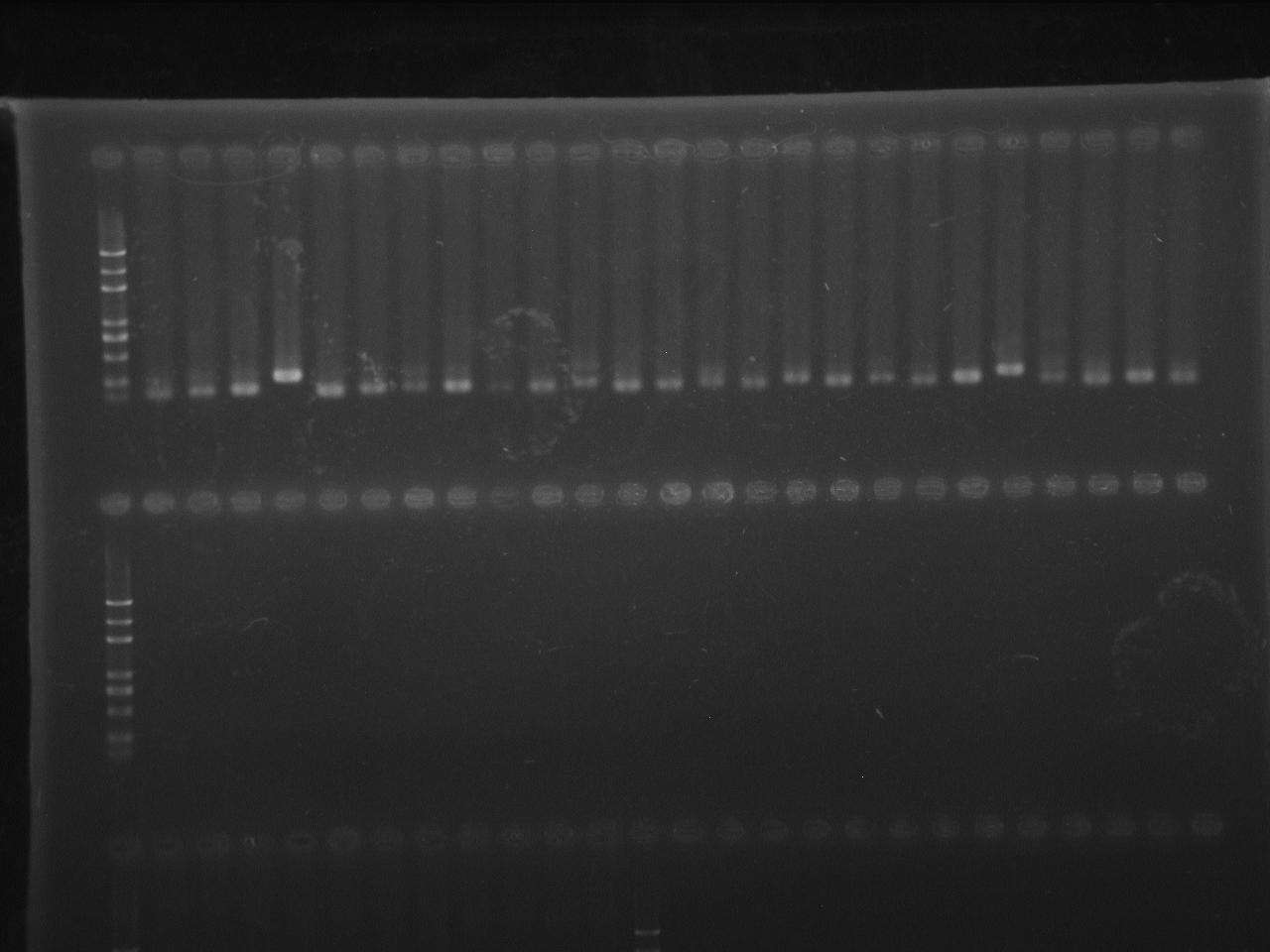

Supplement: Supplemental Information S2 [file peerj-06-5642-s016.zip › The raw photographs, electrophoretic gels and blots-1/Fig. S3 Polymerase chain reaction (PCR) amplification products of all reference genes in L. siceraria leaf in 2% agarose gel-1..png]

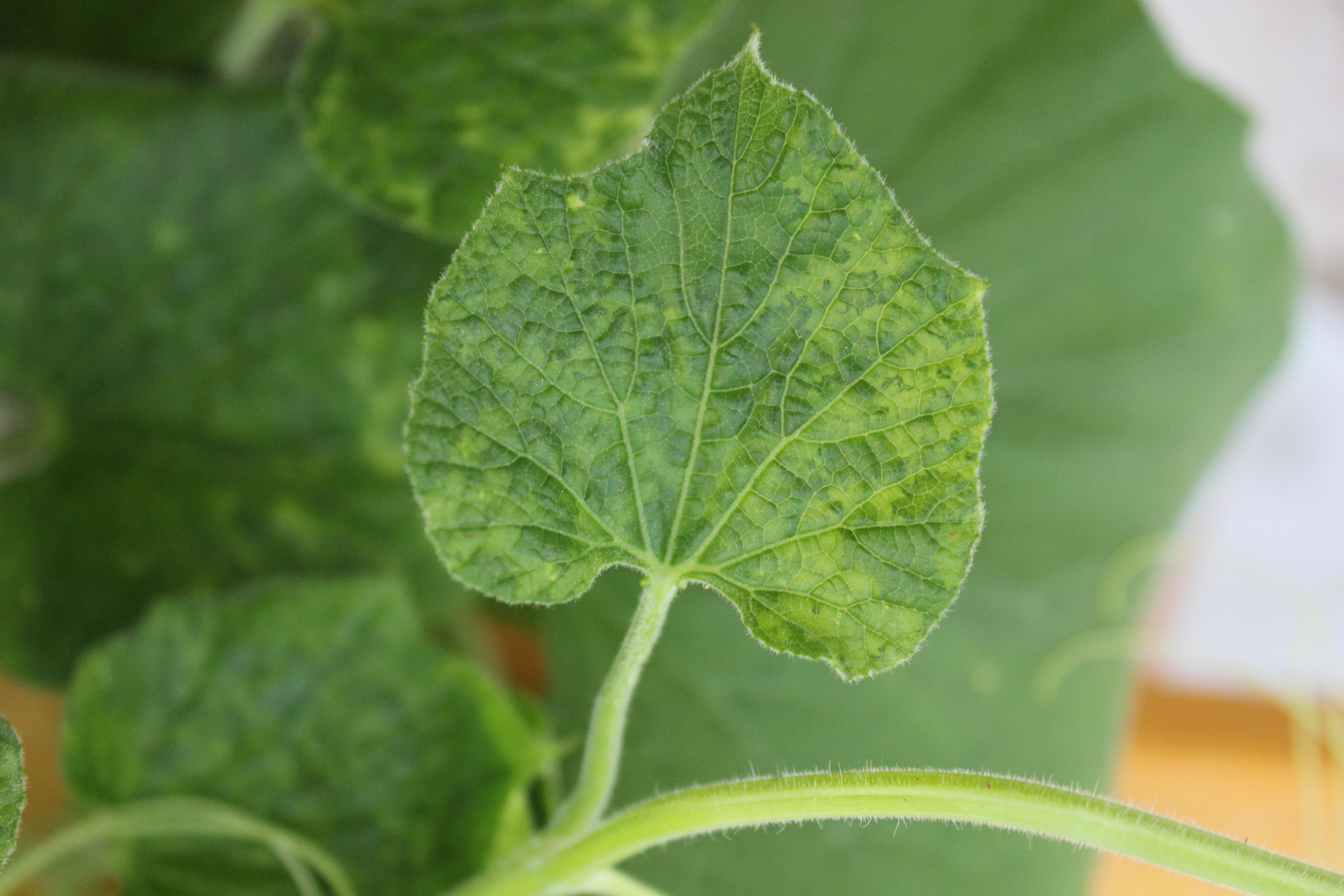

Supplement: Supplemental Information S3 [file peerj-06-5642-s017.zip › The raw photographs, electrophoretic gels and blots-2/Fig. S1 The bottle gourd leaf infected by CGMMV.png]

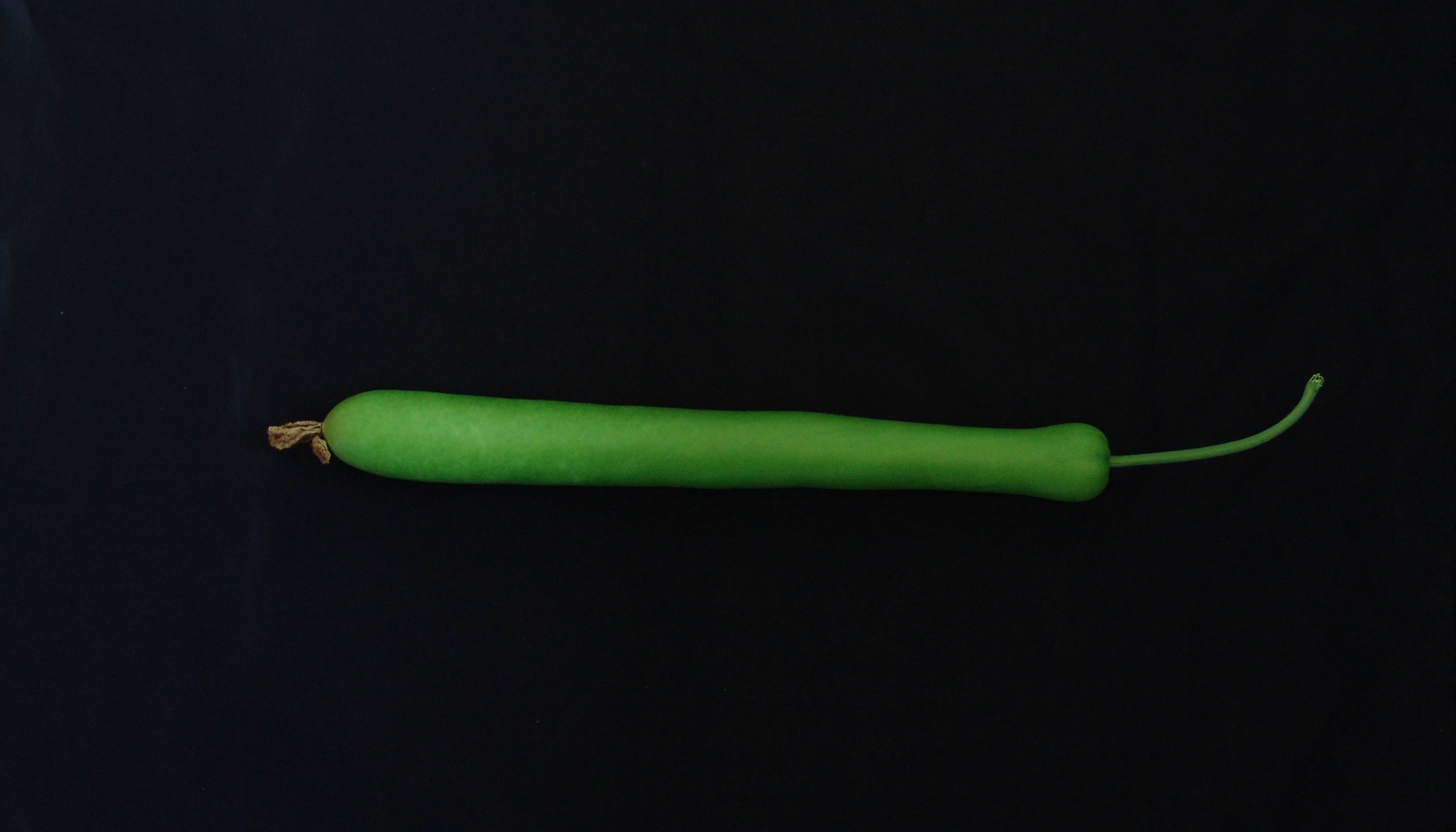

Supplement: Supplemental Information S4 [file peerj-06-5642-s018.zip › The raw photographs, electrophoretic gels and blots-3/Fig. S1 The bottle gourd fruit infected by CGMMV.png]

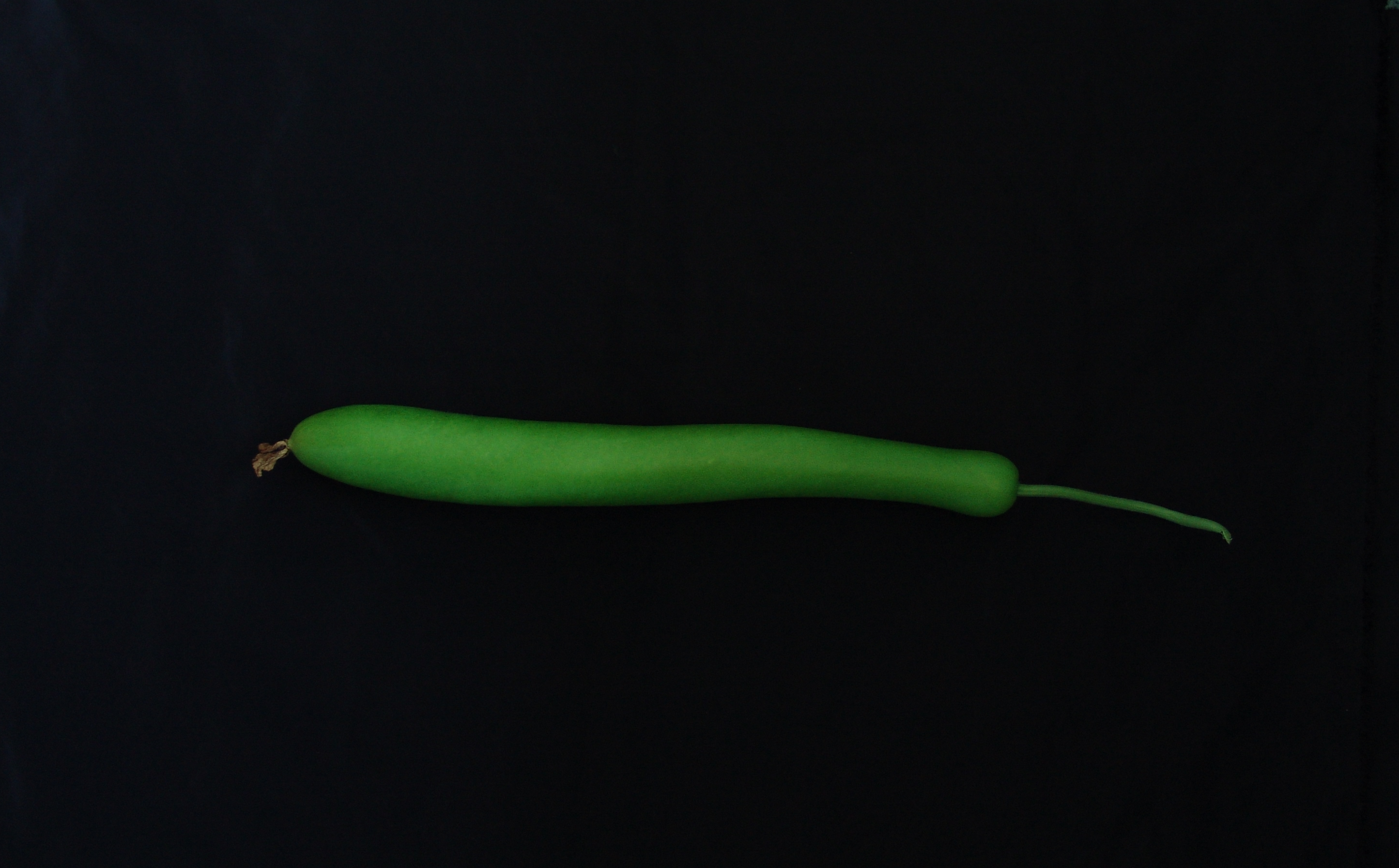

Supplement: Supplemental Information S4 [file peerj-06-5642-s018.zip › The raw photographs, electrophoretic gels and blots-3/Fig. S1 The healthy control bottle gourd fruit.png]
