# Supplementary material for: Genome-wide identification of new reference genes for RT-qPCR normalization in CGMMV-infected Lagenaria siceraria
Source: PeerJ. 2018 Oct 12;6:e5642. doi: 10.7717/peerj.5642 (PMC6188008; doi:10.7717/peerj.5642)
Supplement: Table S6 [file peerj-06-5642-s011.docx]

**Table S6 The results of the transcriptome data screening from fourteen different of cucurbitaceous crops.**

| **Gene symbol** | **transcript_id(s)** | **Number of transcripts** | **The leaves of gourd** | | | | | **The fruits of gourd** | | | | |
| --- | --- | --- | --- | --- | --- | --- | --- | --- | --- | --- | --- | --- |
|  |  |  | **rpkm max** | **rpkm min** | **max/min** | **CV** | **pvalue** | **rpkm max** | **rpkm min** | **max/min** | **CV** | **pvalue** |
| *LsACT* | comp16983_c0_seq1 | 1 | 619.65 | 260.75 | 2.38 | 0.35 | 0.39 | 1973.20 | 904.78 | 2.18 | 0.33 | 0.02 |
| *LsCYP20* | comp4293_c0_seq1 | 1 | 114.04 | 51.05 | 2.23 | 0.30 | 0.24 | 317.73 | 98.95 | 3.21 | 0.41 | 0.01 |
| ***LsEF1α*** | comp17004_c0_seq1 | 1 | **1003.32** | **601.58** | **1.67** | **0.21** | **0.10** | 1758.15 | 767.92 | 2.29 | 0.27 | 0.12 |
| *LsGAPDH* | comp5093_c1_seq1 | 1 | 10.28 | 4.88 | 2.11 | 0.26 | 0.18 | **15.15** | **10.17** | **1.49** | **0.15** | **0.75** |
| ***LsPP2A*** | comp12607_c0_seq1 | 3 | **106.27** | **77.09** | **1.38** | **0.13** | **0.56** | **202.48** | **125.57** | **1.61** | **0.16** | **0.08** |
| *LsRAN* | comp4285_c0_seq1 | 1 | 364.49 | 81.13 | 4.49 | 0.52 | 0.41 | 164.27 | 76.56 | 2.15 | 0.29 | 0.02 |
| ***LsRPS15*** | comp17885_c0_seq1 | 1 | **107.92** | **56.47** | **1.91** | **0.21** | **0.39** | 194.28 | 60.98 | 3.19 | 0.36 | 0.04 |
| *LsTUA* | comp16105_c0_seq5 | 8 | 1725.93 | 603.77 | 2.86 | 0.38 | 0.29 | **4867.59** | **3084.27** | **1.58** | **0.19** | **0.77** |
| ***LsCYP*** | comp17066_c0_seq1 | 1 | **860.08** | **573.74** | **1.50** | **0.14** | **0.66** | 631.57 | 219.13 | 2.88 | 0.34 | 0.02 |
| ***LsRPL23*** | comp15323_c0_seq1 | 6 | 140.56 | 76.72 | 1.83 | 0.20 | 0.44 | 244.65 | 82.39 | 2.97 | 0.31 | 0.12 |
| ***LsADP*** | comp17106_c0_seq1 | 1 | **405.12** | **294.51** | **1.38** | **0.11** | **0.74** | **1118.41** | **619.87** | **1.80** | **0.23** | **0.00** |
| *LsUBA52* | comp17108_c0_seq1 | 1 | 405.03 | 189.41 | 2.14 | 0.26 | 0.44 | 743.24 | 292.73 | 2.54 | 0.27 | 0.15 |
| ***LsTBP*** | comp16607_c0_seq1 | 6 | **52.57** | **38.38** | **1.37** | **0.32** | **0.80** | **66.17** | **55.07** | **1.20** | **0.12** | **0.25** |
| *LsL23A* | comp17854_c0_seq1 | 1 | 148.34 | 66.49 | 2.23 | 0.25 | 0.80 | 174.47 | 60.51 | 2.88 | 0.09 | 0.22 |
