# Supplementary material for: Genome-wide identification of new reference genes for RT-qPCR normalization in CGMMV-infected Lagenaria siceraria
Source: PeerJ. 2018 Oct 12;6:e5642. doi: 10.7717/peerj.5642 (PMC6188008; doi:10.7717/peerj.5642)
Supplement: Table S7 [file peerj-06-5642-s012.docx]

**Table S7 Ranking of the candidate reference genes in bottle gourd leaves and fruits separately screened from the RNA-Seq dataset according to their stability value using Bestkeeper and RefFinder analysis.**

|  | **leaves** | | |  | **fruits** | | |
| --- | --- | --- | --- | --- | --- | --- | --- |
|  | **Bestkeeper** | | **RefFinder** |  | **Bestkeeper** | | **RefFinder** |
| **Gene name** | **Stability value** | **Ranking order** | **Ranking order** | **Gene name** | **Stability value** | **Ranking order** | **Ranking order** |
| *LsCYP* | 0.995 | 1 | 1 | *LsP4H* | 0.978 | 1 | 1 |
| *LsTBP* | 0.964 | 3 | 3 | *LsVAMP* | 0.975 | 2 | 4 |
| *LsH3* | 0.984 | 2 | 2 | *LsTBP* | 0.959 | 3 | 3 |
| *LsWD* | 0.933 | 7 | 7 | *LsADP* | 0.872 | 5 | 2 |
| *LsARL* | 0.947 | 6 | 6 | *LsYpgQ* | 0.844 | 7 | 8 |
| *LsPP2A* | 0.953 | 4 | 4 | *LsXRN1* | 0.884 | 4 | 5 |
| *LsSRK2I* | 0.947 | 5 | 5 | *LsEIF5* | 0.744 | 9 | 7 |
| *LsSK* | 0.933 | 8 | 10 | *LsISCA* | -- | -- | 9 |
| *LsADP* | -- | -- | 13 | *LsH3* | 0.798 | 8 | 6 |
| *LsPDI* | 0.931 | 9 | 8 | *LsARIA* | -- | -- | 13 |
| *LsTPT* | -- | -- | 11 | *LsWD* | 0.720 | 10 | 10 |
| *LsRNC* | -- | -- | 14 | *LsPARP* | -- | -- | 12 |
| *LsUBC* | 0.870 | 10 | 9 | *LsCRCK3* | -- | -- | 11 |
| *LsGAD* | -- | -- | 12 | *LsPP2A* | -- | -- | 15 |
| *LsCNX* | -- | -- | 15 | *LsclpC* | -- | -- | 16 |
| *LsRPS15* | -- | -- | 16 | *LsGAPDH* | -- | -- | 17 |
| *LsEF1α* | -- | -- | 17 | *LsTUA* | -- | -- | 18 |
| *LsRPL23* | -- | -- | 18 | *LsPL* | -- | -- | 14 |
