# Supplementary material for: Genome-wide identification of new reference genes for RT-qPCR normalization in CGMMV-infected Lagenaria siceraria
Source: PeerJ. 2018 Oct 12;6:e5642. doi: 10.7717/peerj.5642 (PMC6188008; doi:10.7717/peerj.5642)
Supplement: Table S8 [file peerj-06-5642-s013.docx]

**Table S8 The data of *LsIPT* and *LsDdRP* from the RNA-seq database.**

| **Gene symbol** | **Gene name** | **transcript_id(s)** | **length** | **effective_ length** | **L1_LCG_rpkm** | **L2_LCG_rpkm** | **L3_LCG_rpkm** | **L1_LCK_rpkm** | **L2_LCK_rpkm** | **L3_LCK_rpkm** | **p-value** | **log2fold_change** | **FDR** | **significant** |
| --- | --- | --- | --- | --- | --- | --- | --- | --- | --- | --- | --- | --- | --- | --- |
| Leaf-*LsIPT* | adenylate isopentenyltransferase (cytokinin synthase) | comp218659_c0_seq1 | 971 | 971.00 | 1.91 | 2.33 | 1.30 | 0.06 | 1.19 | 0.36 | 0.04 | 1.78 | 0.80 | yes |
| Leaf-*LsDdRP* | DNA-directed RNA polymerase II subunit G | comp19482_c0_seq1 | 835 | 835.00 | 72.04 | 47.26 | 50.30 | 30.04 | 14.61 | 19.58 | 0.03 | 1.40 | 0.80 | yes |
| Fruit-*LsIPT* | adenylate isopentenyltransferase (cytokinin synthase) | comp218659_c0_seq1 | 971 | 971.00 | 0.10 | 0.17 | 0.10 | 0.25 | 0.29 | 0.31 | 0.01 | -1.20 | 0.07 | yes |
| Fruit-*LsDdRP* | DNA-directed RNA polymerase II subunit G | comp19482_c0_seq1 | 835 | 835.00 | 100.50 | 92.54 | 98.09 | 28.23 | 37.08 | 28.78 | 0.00 | 1.63 | 0.02 | yes |
