# Supplementary material for: Genome-wide identification of new reference genes for RT-qPCR normalization in CGMMV-infected Lagenaria siceraria
Source: PeerJ. 2018 Oct 12;6:e5642. doi: 10.7717/peerj.5642 (PMC6188008; doi:10.7717/peerj.5642)
Supplement: Supplemental Information S1 [file peerj-06-5642-s015.zip › Detection result of leaf and fruit variation in bottle gourd/SNP/Description.docx]

Description of each file under the SNP Muscle folder:

>Gene name_Sample name_ Mutation site Ref=>ALT

In the sequence content: （ALT//Ref）
