# Supplementary material for: Genome-wide identification of new reference genes for RT-qPCR normalization in CGMMV-infected Lagenaria siceraria
Source: PeerJ. 2018 Oct 12;6:e5642. doi: 10.7717/peerj.5642 (PMC6188008; doi:10.7717/peerj.5642)
Supplement: Supplemental Information S2 [file peerj-06-5642-s016.zip › The raw photographs, electrophoretic gels and blots-1/The raw photographs, electrophoretic gels and blots were all photographed by the Chenhua Zhang..docx]

The raw photographs, electrophoretic gels and blots were all photographed by the Chenhua Zhang.
